# Multicohort and cross‐platform validation of a prognostic Wnt signature in colorectal cancer

**DOI:** 10.1002/ctm2.199

**Published:** 2020-12-29

**Authors:** Frauke Goeman, Francesca De Nicola, Carla Azzurra Amoreo, Stefano Scalera, Daniele Marinelli, Francesca Sperati, Marco Mazzotta, Irene Terrenato, Matteo Pallocca, Ludovica Ciuffreda, Eleonora Sperandio, Maddalena Barba, Laura Pizzuti, Domenico Sergi, Antonella Amodio, Giancarlo Paoletti, Eriseld Krasniqi, Patrizia Vici, Beatrice Casini, Enzo Gallo, Simonetta Buglioni, Maria Grazia Diodoro, Edoardo Pescarmona, Ilio Vitale, Ruggero De Maria, Gian Luca Grazi, Gennaro Ciliberto, Maurizio Fanciulli, Marcello Maugeri‐Saccà

**Affiliations:** ^1^ Oncogenomic and Epigenetic Unit IRCCS “Regina Elena” National Cancer Institute Rome Italy; ^2^ SAFU Laboratory, Department of Research, Advanced Diagnostic, and Technological Innovation IRCCS “Regina Elena” National Cancer Institute Rome Italy; ^3^ Department of Pathology IRCCS “Regina Elena” National Cancer Institute Rome Italy; ^4^ Medical Oncology Unit, Department of Clinical and Molecular Medicine, Sant'Andrea Hospital “Sapienza” University of Rome Rome Italy; ^5^ Biostatistics Unit San Gallicano Dermatological Institute IRCCS Rome Italy; ^6^ Division of Medical Oncology 2 IRCCS “Regina Elena” National Cancer Institute Rome Italy; ^7^ Biostatistics‐Scientific Direction IRCCS “Regina Elena” National Cancer Institute Rome Italy; ^8^ Italian Institute for Genomic Medicine (IIGM) IRCSS Candiolo Turin Italy; ^9^ Candiolo Cancer Institute FPO ‐ IRCCS Turin Italy; ^10^ Institute of General Pathology Catholic University of the Sacred Heart Rome Italy; ^11^ Institute of General Pathology IRCCS Fondazione Policlinico Universitario Agostino Gemelli Rome Italy; ^12^ Hepato‐Pancreato‐Biliary Surgery IRCCS “Regina Elena” National Cancer Institute Rome Italy; ^13^ Scientific Direction IRCCS “Regina Elena” National Cancer Institute Rome Italy

Dear Editor,

Deregulation of the Wnt pathway is a hallmark of colorectal cancer (CRC). Nevertheless, the clinical implications of aberrant β‐catenin‐driven gene transcription remain elusive.^1^

We herein present a transcriptional WNT signature predicting survival outcomes in metastatic CRC (mCRC). Wnt was studied in a discovery cohort (94 mCRC patients treated with first‐line therapy at Regina Elena National Cancer Institute, IRE cohort, Table S1) by combining targeted RNA‐Seq (expression levels of 93 Wnt‐associated genes, Illumina TruSeq Targeted RNA Expression Wnt Panel) and targeted DNA sequencing.^2^ Given the molecular communication between Wnt and the DNA damage repair (DDR) system,^3^ as well as the Hippo pathway,^4^ immunohistochemistry (IHC) in tissue microarrays (TMAs) was used for studying protein‐level markers (pRPA32, pATR, pCHK1, pWEE1, γH2AX, pATM, pCHK2, YAP, and TAZ).

For external validation, we considered three independent and publically available datasets (N = 1366) previously used for developing the consensus molecular subtype^5^: TCGA, N = 576 (RNA‐Seq)^6^; GSE39582, N = 558,^7^ and GSE17538, N = 232 (Affymetrix microarrays).^8^

In our analytical workflow, eight genes were selected relying on their association with survival outcomes (progression‐free survival [PFS], overall survival [OS], and best overall response [BOR]). These genes were combined into a transcriptional signature exploiting their co‐expression pattern: Wnt (+) tumors were defined as tumors with overexpression of at least four genes, whereas those samples that did not fulfill this criterion were defined as Wnt (–) (0‐3 overexpressed genes). Individual transcripts were considered as high and low using the highest tertile as the cutoff point, even though for exploratory analyses (IRE cohort) the median value was also evaluated. Immunogenomic features were investigated through the CRI iAtlas Portal.^9^ The workflow of this study is illustrated in Figure S1.

In the IRE cohort, we first performed an unsupervised hierarchical clustering that included the entire set of transcripts and the mutational status of Wnt genes (Figure 1A). Given that we did not observe any clear clustering, clinically focused differential gene expression analyses were performed to identify differences between outlier patients (fast vs slow progressors, short‐ vs long‐term survivors, and good vs poor responders). (Figures 1B and S2). Afterward, differentially expressed genes were tested for their relationship with the respective clinical outcome (Figure 1C and 1D). On this basis, eight genes were selected to generate a transcriptional signature (APC, KREMEN, SFRP1, SFRP2, CSNK1A1, PRICKLE1, SOX17, and DKK1).

**FIGURE 1:**
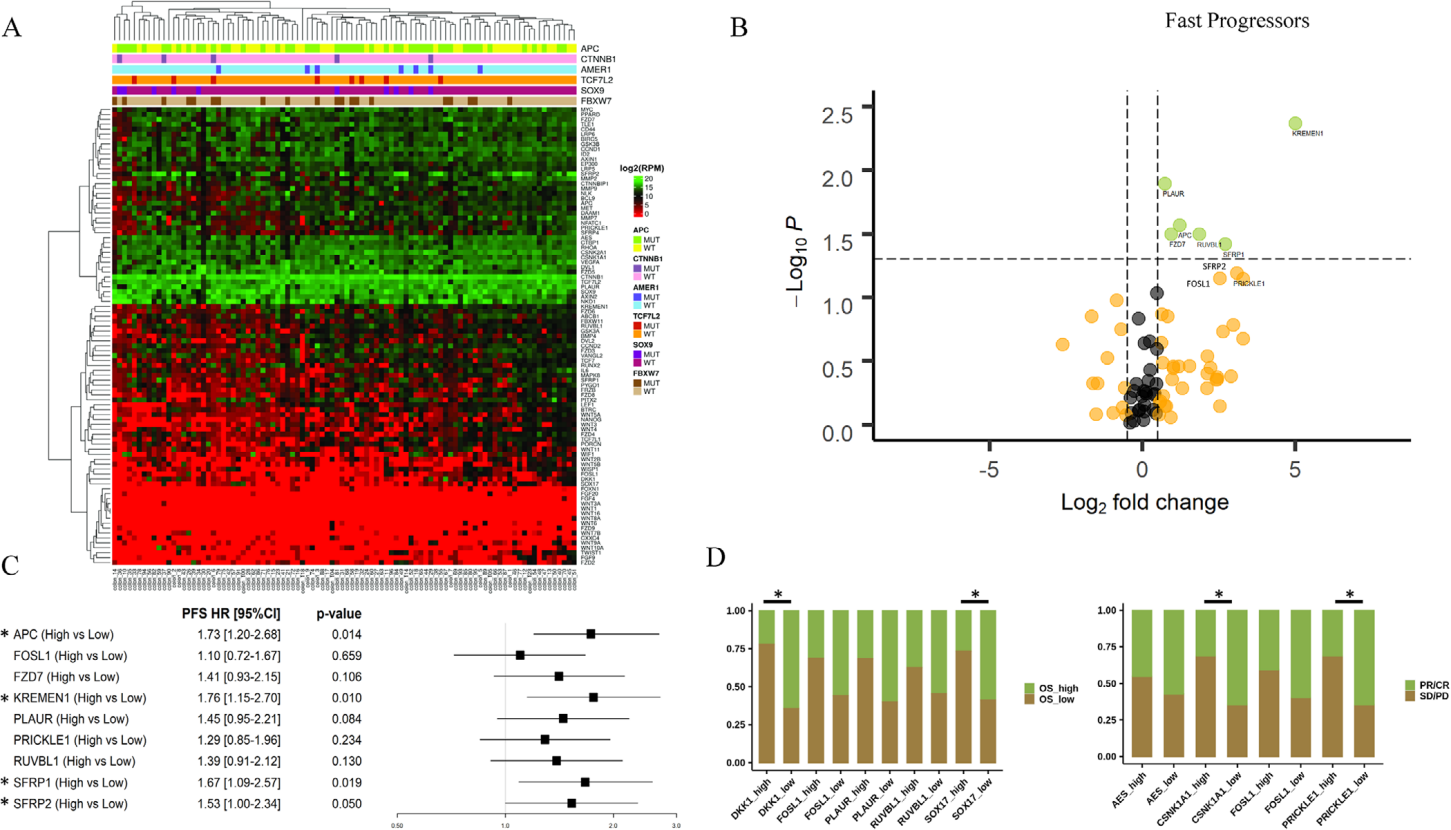
**A,** Heatmap showing unsupervised clustering analysis of Wnt genes in the IRE cohort. Targeted DNA‐ and RNA‐Seq were employed to evaluate the mutational status of six Wnt pathway components (*APC*, *CTNNB1*, *AMER1*, *TCF7L2*, *FBXW7*, and *SOX9*) along with the expression of 93 WNT pathway genes. Genes: rows; tissue samples: columns. **B,** Volcano plot of Wnt genes differently expressed when comparing fast and slow progressors. **C,** Forest plot illustrating univariate Cox regression analyses for progression‐free survival (PFS). **D,** Bar charts illustrating the distribution of individual genes in the highest/lowest quartile of overall survival (left) and in responders/non‐responders (right). Transcripts are classified as high and low using the highest tertile as cutoff point. Asterisks indicate statistical significance (univariate Cox regressions in panel C and Chi2 test in panel D) Abbreviations: CR, complete response; OS, overall survival; PD, progressive disease; PR, partial response; SD, stable disease.

Except for tumor sidedness, we did not record any significant association between the Wnt signature and baseline characteristics of the patients in the IRE cohort (Figure 2A). The Wnt (+) model was significantly overrepresented in rapidly progressing tumors (Figure 2B), and patients with Wnt (+) tumors had significantly shorter PFS and OS as compared to their negative counterparts (log‐rank *P* = .002 and *P* = .003, respectively; Figures 2C and 2D). Multivariate Cox regression models demonstrated that the Wnt (+) signature is an independent predictor (Figure S3). The robustness of the model was confirmed in the TCGA cohort, where patients with Wnt (+) tumors had a significant shorter survival than their negative counterparts (log rank *P* = .017; Figure 3A; multivariate Cox regression model presented in Figure S4).

**FIGURE 2:**
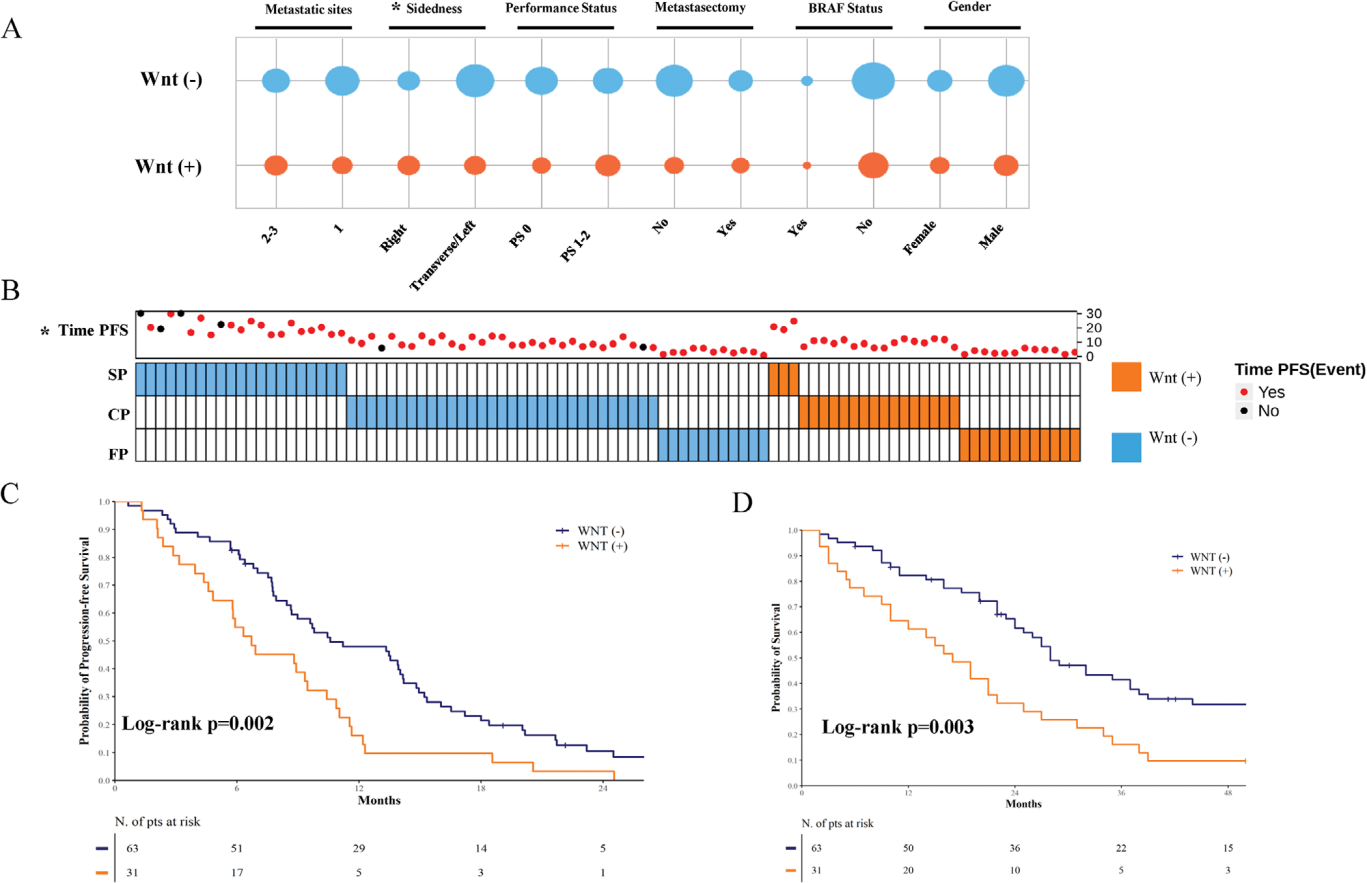
**A,** Bubble chart illustrating the associations between the Wnt signature and standard clinical‐pathological features of mCRC patients included in the IRE cohort. **B,** Oncoprint illustrating the distribution of the Wnt signature according to the three different patterns of disease progression. SP: slow progressors (highest quartile of PFS), CP: conventional progressors (intermediate quartiles of PFS), FP: fast progressors (lowest quartile of PFS). Asterisks in panels **A** and **B** indicate statistical significance (*χ* ^2^, *P* < .05). **C** and **D,** Kaplan‐Meier survival curves of progression‐free survival (PFS) and overall survival (OS) in the IRE cohort

**FIGURE 3:**
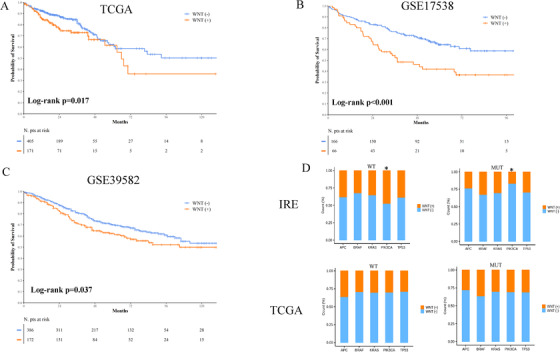
Kaplan‐Meier survival curves of overall survival (OS) in the TCGA (**A**), GSE17538 (**B**), and GSE39582 (**C**) studies. **D,** Bar charts illustrating the distribution of the Wnt signature according to the mutational status of *APC*, *TP53*, *KRAS*, *PIK3CA*, and *BRAF* in the IRE and TCGA studies. Asterisk indicates statistical significance (*χ* ^2^, *P* < .05)

In microarray‐based gene expression studies (GSE17538 and GSE39582), patients with Wnt (+) disease had shorter survival (log‐rank *P* < .001 and *P* = .037, respectively) (Figures 3B and 3C). Notably, we used largely overlapping probesets selected on the basis of the Pearson correlation coefficient (Figure S5). In order to better estimate the connection between the model and OS, survival analysis was carried out in a metadataset containing the TCGA, GSE17538, and GSE39582 studies (log‐rank *P* < .001; Figure S6).

Regarding the connection between our signature and driver mutations (*APC*, *TP53*, *KRAS*, *PI3KCA*, and *BRAF*, available in the IRE and TCGA cohorts), we did not observe any clear association, with the exception of *PIK3CA* mutations in the IRE cohort (Figure 3D).

As aforementioned, protein‐level biomarkers related to the DDR and Hippo pathways have been evaluated in our IRE cohort (IHC on TMAs; Figure S7). The expression levels of DDR markers were similar between Wnt (+) and WNT (–) cases (Figure S8A). Consistently, in the TCGA study neither microsatellite instability nor the homologous repair deficiency signature was associated with Wnt (+) tumors (Figures S8B and S8C). To a similar extent, protein‐level expression of YAP/TAZ and mRNA expression levels of the YAP/TAZ target genes BIRC5 and CCND1 (genes included in our targeted RNA‐Seq panel) were comparable between WNT (+) and WNT (–) tumors (Figures S8D and S8E).

Finally, we investigated a possible link between the Wnt signature and core immune signatures used for generating the immune subtyping of cancers.^9^ Differences were recorded between WNT (+) and WNT (–) tumors for all the tested immune‐related features (Figure 8F), suggesting a different immunological background.

This is the first report describing a multi‐level and clinically focused analysis of the pathway and cross‐talking networks. Two earlier reports supported a prognostic/predictive role for *APC* mutations exclusively within the frame of specific genomic contexts.^2^, ^10^ We acknowledge the intrinsic limitation of a retrospective design. Nevertheless, we analyzed a significant number of patients, and exploiting an analytical workflow conceived to take into account three relevant clinical endpoints (PFS, OS, and BOR).

Our results suggest that (a) the co‐expression pattern of eight Wnt genes identifies mCRC undergoing an unexpectedly rapid disease progression, (b) the relationship between the signature and survival outcomes seems unrelated to common genomic alterations, mechanisms that protect the mammalian genome from genotoxic cues, and YAP/TAZ, and (c) WNT (+) and WNT (–) tumors plausibly harbor a different immunological repertoire.

Collectively, our data indicate that the transcriptional WNT signature holds the potential to predict survival outcomes in mCRC. Moreover, the reproducibility of our findings in four independent studies leveraging two different gene expression technologies (RNA‐Seq and microarrays) suggests the robustness of the signature.

## FUNDING INFORMATION

This study was supported by an intramural research grant to the “Gastrointestinal Tumors Translational Research Group” (MM‐S and MF). MM‐S is supported by a Young Investigator Grant (GR Italian Minister of Health) and My First AIRC Grant (AIRC, Associazione Italiana per la Ricerca sul Cancro).

## AUTHOR CONTRIBUTIONS

MM‐S, GC, MF, IV, and RDM conceived and designed the study. FG, FDN, and LC carried out RNA‐Seq and DNA‐sequencing. MP, FS, IT, SS, ES, and MB performed bioinformatic and statistical analyses. LP, DS, AA, GP, BC, EG, CAA, PV, SB, MGD, EP, EK, DM, MM, and GLG acquired and reviewed clinical and pathological data. All authors have been involved in drafting the manuscript. MM‐S wrote the final version of the manuscript. All authors read and approved the final version of the manuscript and agree to be accountable for all aspects of the work.

## CONFLICT OF INTERESTS

LP received travel grants from Eisai, Roche, Pfizer, and Novartis and speaker fees from Roche, Pfizer, Novartis, and Gentili. DS received travel grants from Roche, Pharma Mar, and Astra Zeneca and personal fees from Roche. PV received travel grants from Eisai, Roche, Pfizer, and Novartis; speaker fees/advisory boards from Roche, Pfizer, Novartis, and Gentili. RDM declares to be a scientific advisory board member at Exosomics SpA (Siena IT), Hibercell Inc (New York, NY, USA), Kiromic Inc (Houston, TX, USA), and at Exiris Inc (Rome, Italy). The other authors declare no conflict of interest.

## Supporting information

Table S1. Baseline characteristics of metastatic colorectal cancer (mCRC) patients included in the IRE cohort (N = 94).

Figure S1. Flow diagram of the study. Abbreviations: mCRC: metastatic colorectal cancer, IHC: immunohistochemistry, TMAs: tissue microarrays.

Figure S2. Volcano plots of Wnt genes differently expressed when comparing long‐term versus short‐term survival (highest and lowest quartile, respectively) (A) and responders versus nonresponders (B) in the IRE cohort. Abbreviations: CR: complete response, OS: overall survival, PD: progressive disease, PR: partial response, SD: stable disease.

Figure S3. Forest plots illustrating multivariate Cox regression analyses for progression‐free survival (PFS) (A) and overall survival (OS) (B) in the IRE cohort.

Figure S4. Forest plot illustrating the multivariate Cox regression analysis for overall survival (OS) in the TCGA study.

Figure S5. Heatmaps illustrating the Pearson correlation coefficient of probesets representing the genes of interest in the GSE39582 (A) and GSE17538 (B) studies. Asterisks indicate the probesets selected for generating the model in microarray studies.

Figure S6. Kaplan‐Meier survival curves of overall survival (OS) in a metadataset consisting of the TCGA, GSE39582 and GSE17538 cohorts.

Figure S7. Representative examples of immunohistochemical expression (tissue microarrays, TMAs) of DNA damage response (DDR) markers (γH2AX, pATM, pCHK2, pRPA32, pATR, pCHK1, and pWEE1) (A) and YAP and TAZ (IRE cohort) (B).

Figure S8. A, Heatmap illustrating the expression levels of phosphorylated DDR markers in Wnt (+) and Wnt (–) tumors in the IRE cohort. The expression levels of DDR markers were calculated by multiplying staining intensity (0‐3) X the percentage of nuclear‐expressing tumor cells (final score range: 0‐300). B, Stacked bar chart summarizing the distribution of CRC TCGA subtypes in Wnt (+) and Wnt (–) cases. CIN: chromosomal instability, GS: genomically stable, MSI/POLE microsatellite instability/POLE. C, Violin plot illustrating the homologous repair deficiency (HRD) signature enrichment score in Wnt (+) and Wnt (–) cases in the TCGA study. D, Heatmap showing protein‐level expression of the Hippo transducers YAP and TAZ (IRE cohort) in the Wnt (+) and Wnt (–) groups. The expression of YAP/TAZ was calculated with the same method used for DDR markers. E, Box plot for the YAP/TAZ target genes BIRC5 and CCND1 (IRE cohort). NS: not significant. f Violin plots illustrating core immune signature scores (and the auxiliary proliferation signature) in the two compared subgroups (TCGA study). In the violin plots, NS: not significant, *: Mann‐Whitney test *P* < .05.

## Data Availability

The data that support the findings of this study are available from the corresponding author upon reasonable request.
