# Supplementary material for: Multicohort and cross‐platform validation of a prognostic Wnt signature in colorectal cancer
Source: Clin Transl Med. 2020 Dec 29;10(8):e199. doi: 10.1002/ctm2.199 (PMC7770515; doi:10.1002/ctm2.199)
Supplement: Supplementary file 1 — Table S1. Baseline characteristics of metastatic colorectal cancer (mCRC) patients included in the IRE cohort (N = 94). [file CTM2-10-e199-s009.doc]

**Supplementary Table 1**: Baseline characteristics of metastatic colorectal cancer (mCRC) patients included in the IRE cohort (N=94).

| **Characteristics** |  | **N (%)** |
| --- | --- | --- |
| **Age at diagnosis** | Median [IQ range] | 61.7 [55.8-69.9] |
| **Gender** | Male | 62 (66.0) |
|  | Female | 32 (34.0) |
| **Stage at diagnosis** | II-III | 34 (36.2) |
|  | IV | 60 (63.8) |
| **(Neo)Adjuvant therapy** | No | 68 (72.3) |
|  | Yes | 26 (27.7) |
| **ECOG PS** | 0 | 45 (47.9) |
|  | 1-2 | 49 (52.1) |
| **Side** | Right | 32 (34.0) |
|  | Transverse | 10 (10.6) |
|  | Left | 52 (55.3) |
| **Number of metastatic sites** | 1 | 52 (55.3) |
|  | ≥2 | 42 (44.7) |
| **Surgery for metastatic disease** | No | 65 (69.1) |
|  | Yes | 29 (30.9) |
| **First-line therapy** | Chemotherapy* | 58 (61.7) |
|  | Chemotherapy/Cetuximab** | 20 (21.3) |
|  | Chemotherapy/Bevacizumab*** | 16 (17.0) |
| **Second-line therapy** | No | 30 (31.9) |
|  | Yes | 64 (68.1) |
| **Targeted Agent** | No | 43 (45.7) |
|  | Yes (first-line and beyond) | 51 (54.3) |

*FOLFIRI N=39, FOLFOX N=19.

**FOLFIRI/Cetuximab N=14, FOLFOX/Cetuximab N=5, CPT-11/Cetuximab N=1.

***FOLFIRI/Bevacizumab N=11, FOLFOX/Bevacizumab N=4, Capecitabine/Bevacizumab N=1.
