# Supplementary figures and images for: Multicohort and cross‐platform validation of a prognostic Wnt signature in colorectal cancer
Source: Clin Transl Med. 2020 Dec 29;10(8):e199. doi: 10.1002/ctm2.199 (PMC7770515; doi:10.1002/ctm2.199)

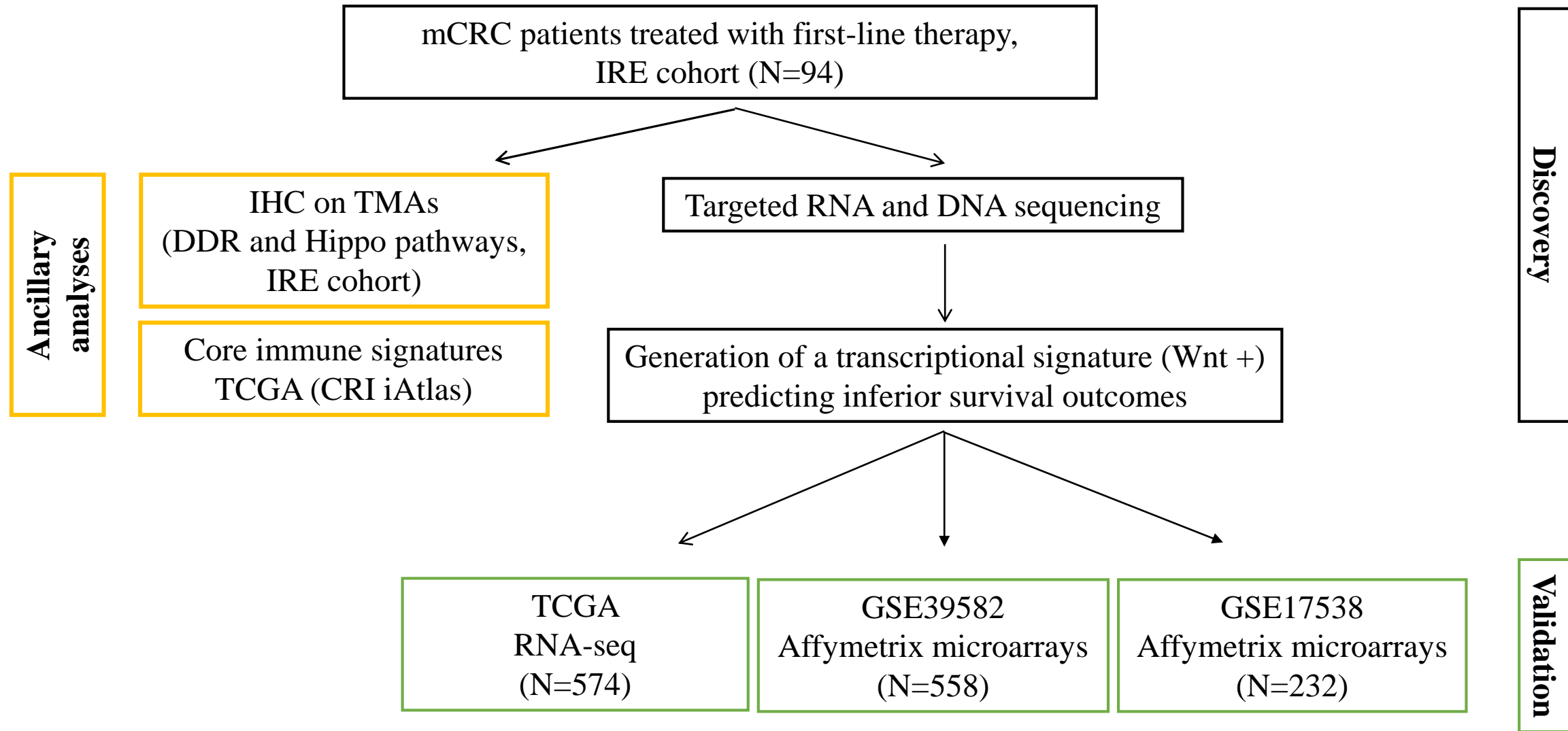

Supplement: Supplementary file 2 — Figure S1. Flow diagram of the study. Abbreviations: mCRC: metastatic colorectal cancer, IHC: immunohistochemistry, TMAs: tissue microarrays. [file CTM2-10-e199-s001.pdf]

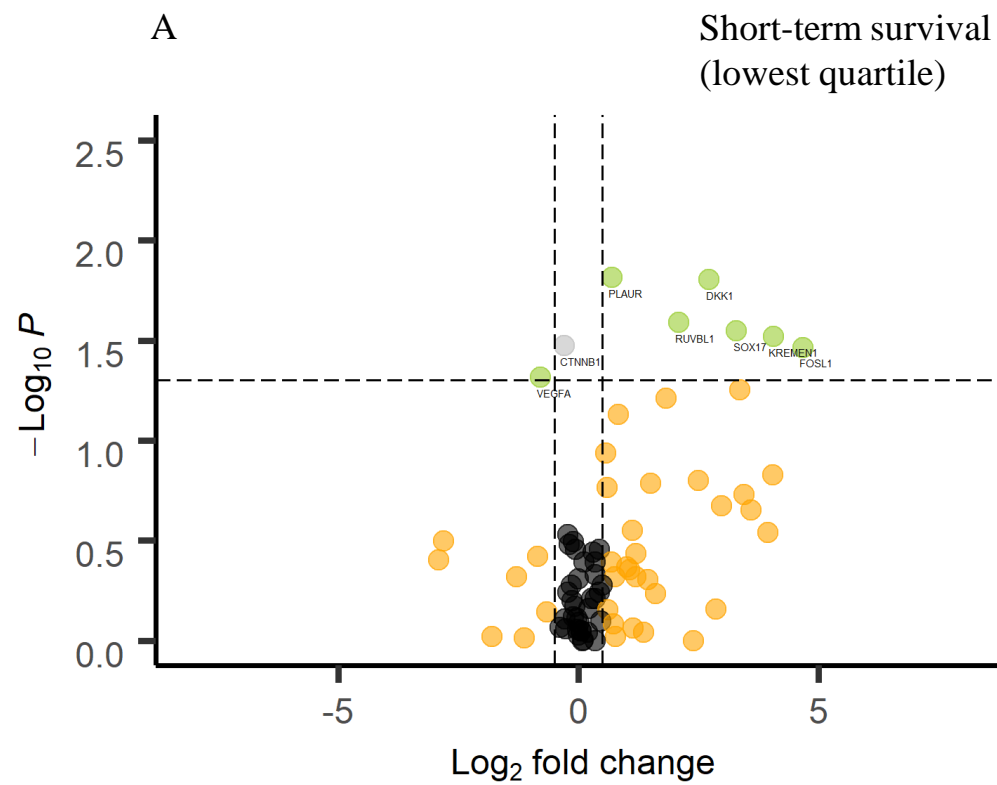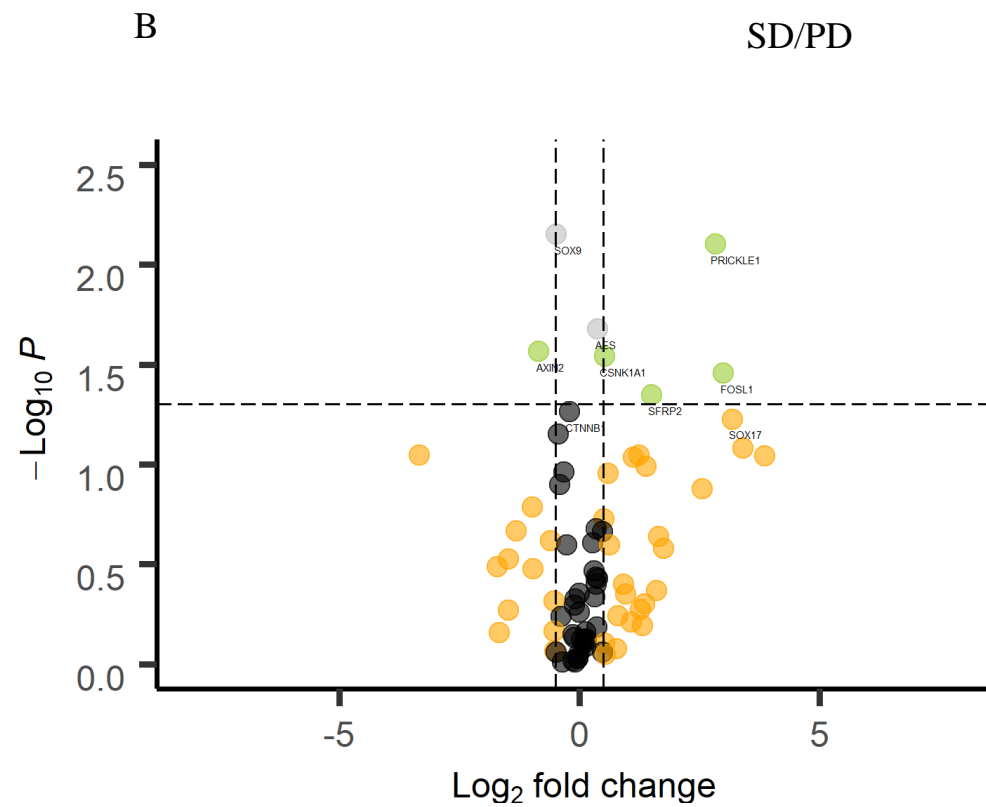

Supplement: Supplementary file 3 — Figure S2. Volcano plots of Wnt genes differently expressed when comparing long‐term versus short‐term survival (highest and lowest quartile, respectively) (A) and responders versus nonresponders (B) in the IRE cohort. Abbreviations: CR: complete response, OS: overall survival, PD: progressive disease, PR: partial response, SD: stable disease. [file CTM2-10-e199-s002.pdf]

A

GSE39582

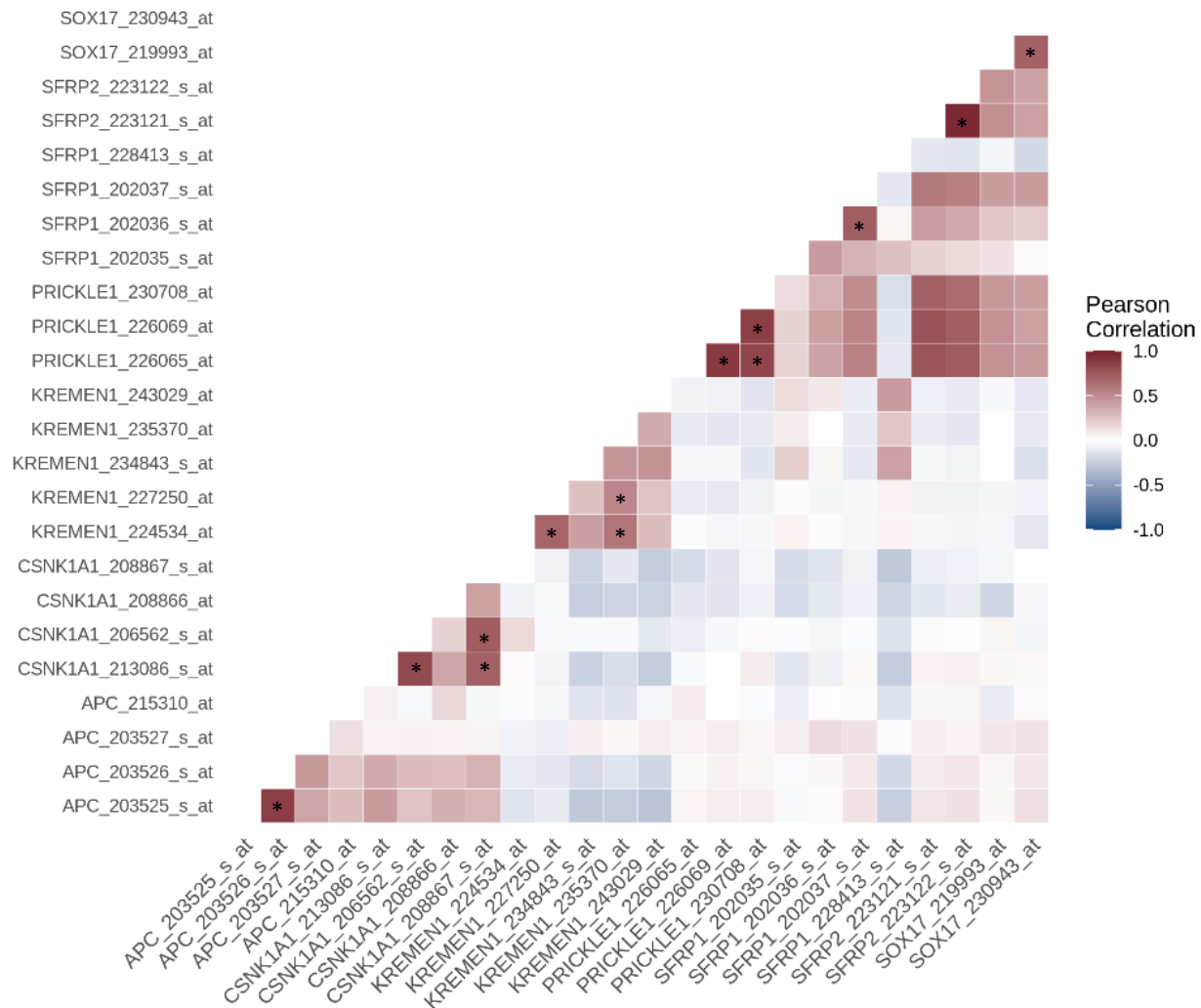

B

GSE17538

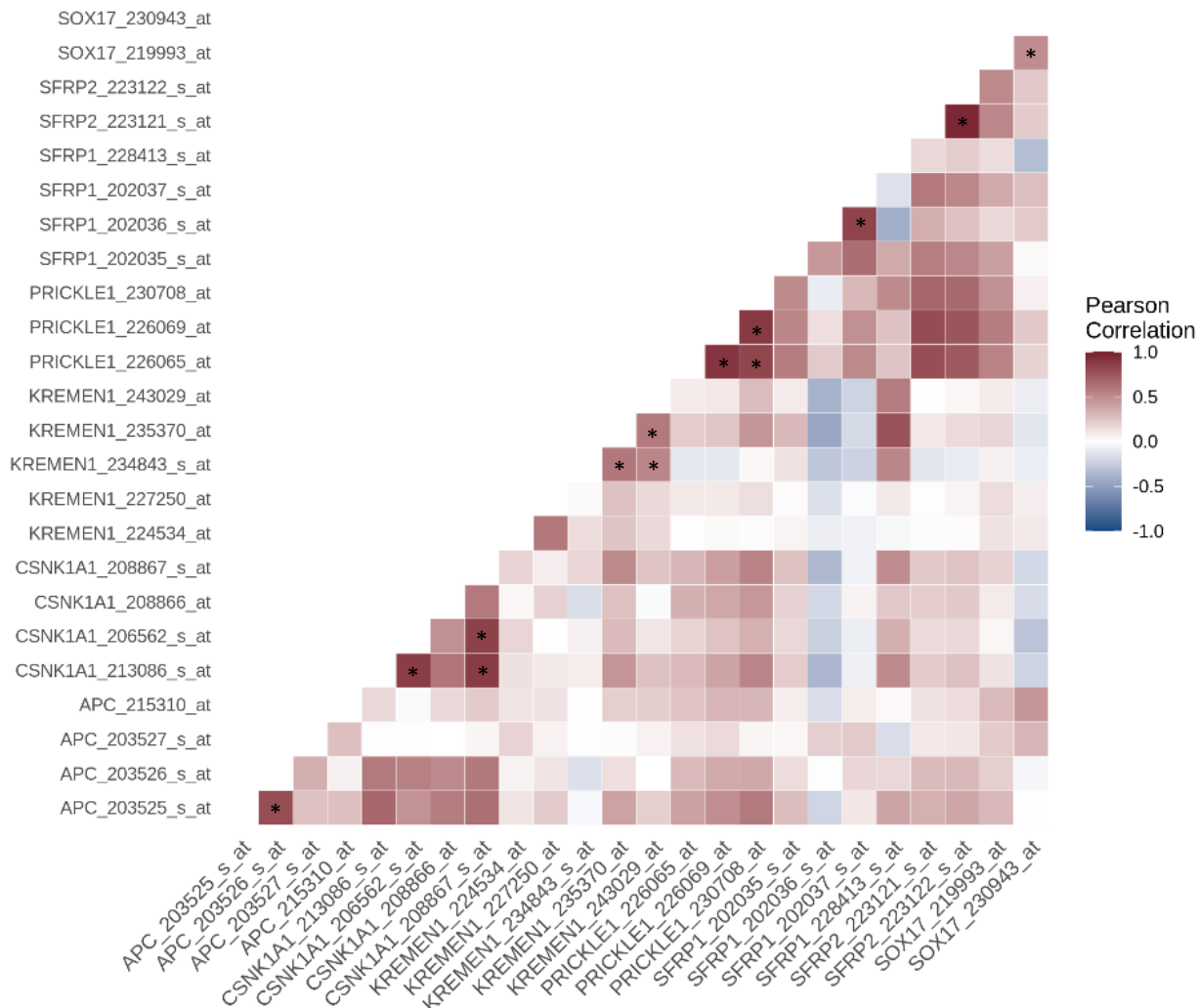

Supplement: Supplementary file 6 — Figure S5. Heatmaps illustrating the Pearson correlation coefficient of probesets representing the genes of interest in the GSE39582 (A) and GSE17538 (B) studies. Asterisks indicate the probesets selected for generating the model in microarray studies. [file CTM2-10-e199-s005.pdf]

# Metadataset (TCGA/GSE17538/GSE39582)

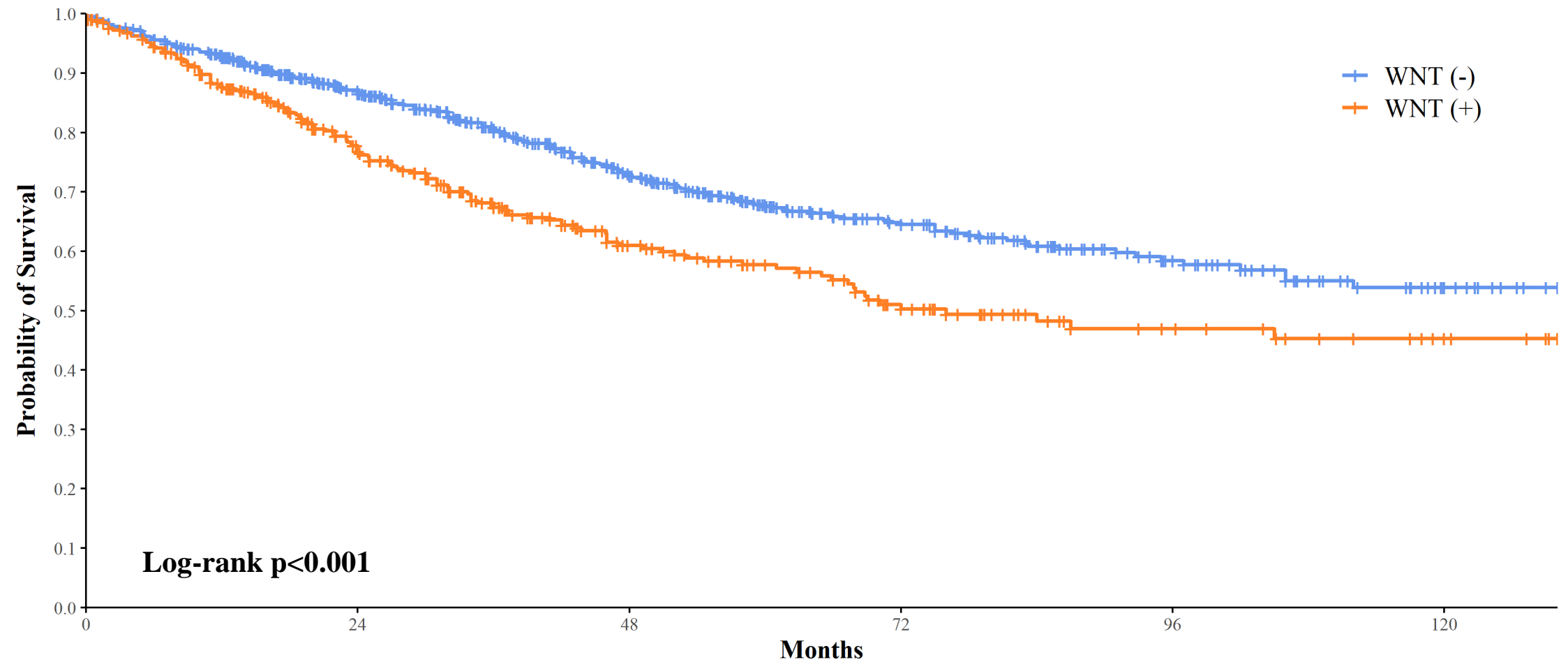

N. pts at risk

|     |     |     |     |    |    |
|-----|-----|-----|-----|----|----|
| 957 | 630 | 364 | 190 | 81 | 39 |
| 409 | 245 | 120 | 67  | 31 | 20 |

Supplement: Supplementary file 7 — Figure S6. Kaplan‐Meier survival curves of overall survival (OS) in a metadataset consisting of the TCGA, GSE39582 and GSE17538 cohorts. [file CTM2-10-e199-s006.pdf]

A

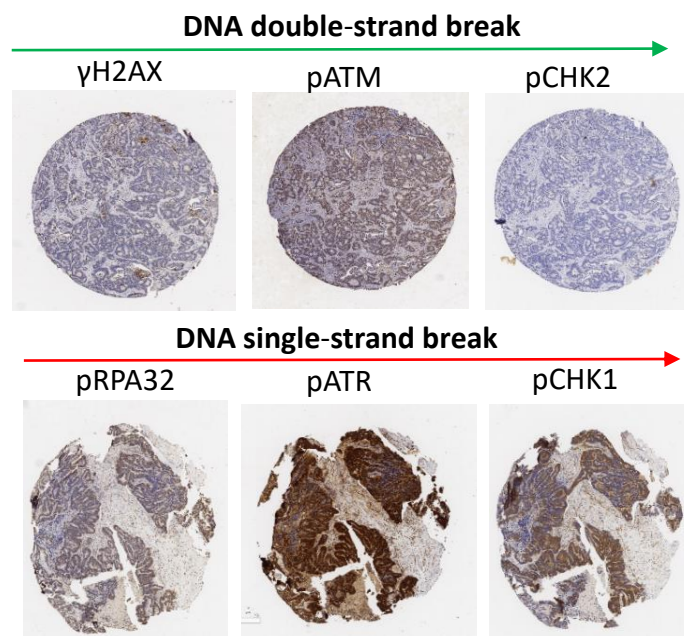

B

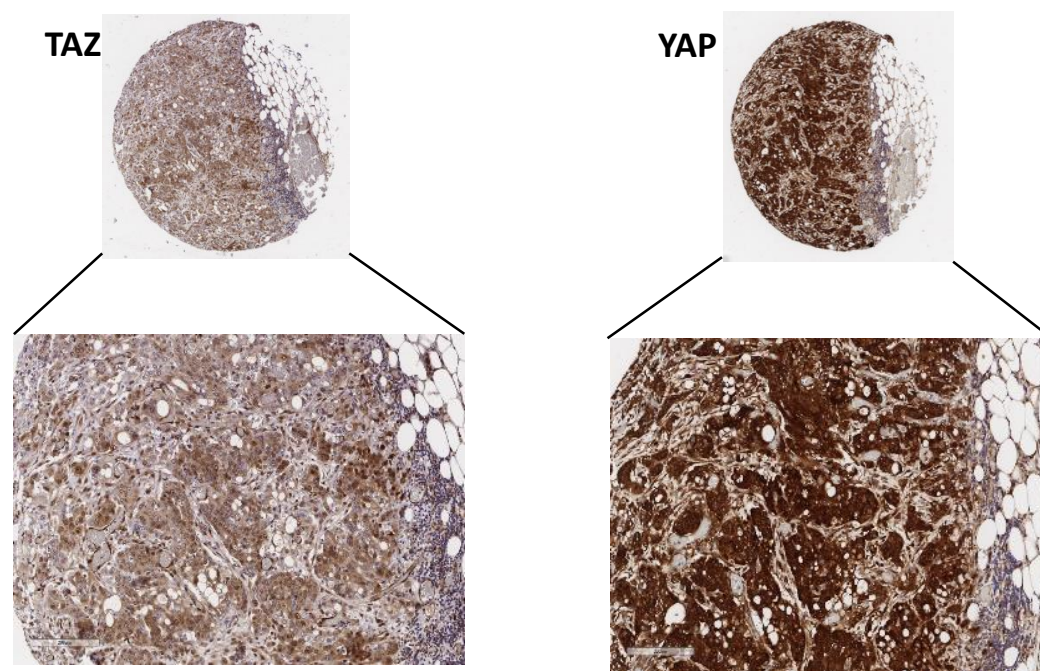

Supplement: Supplementary file 8 — Figure S7. Representative examples of immunohistochemical expression (tissue microarrays, TMAs) of DNA damage response (DDR) markers (γH2AX, pATM, pCHK2, pRPA32, pATR, pCHK1, and pWEE1) (A) and YAP and TAZ (IRE cohort) (B). [file CTM2-10-e199-s007.pdf]

A

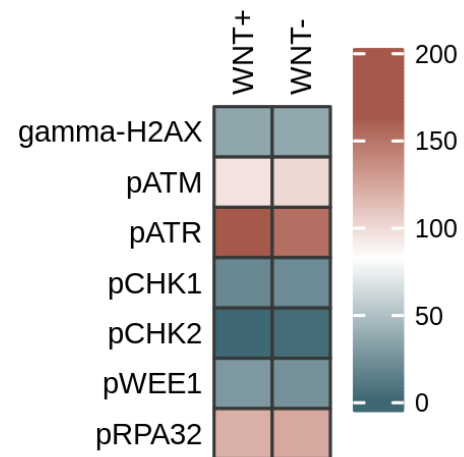

B

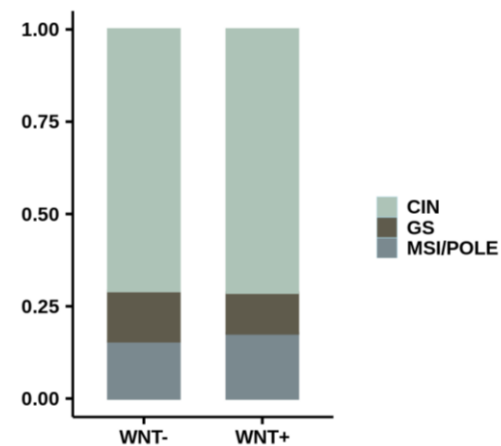

C

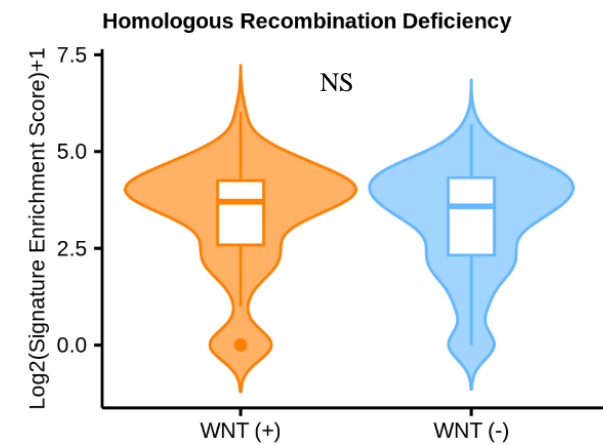

D

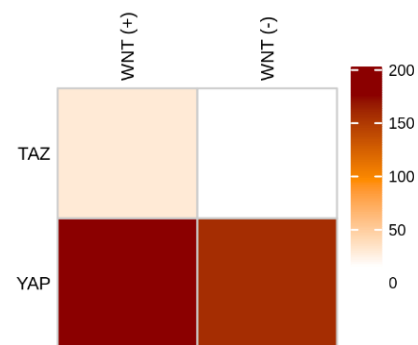

F

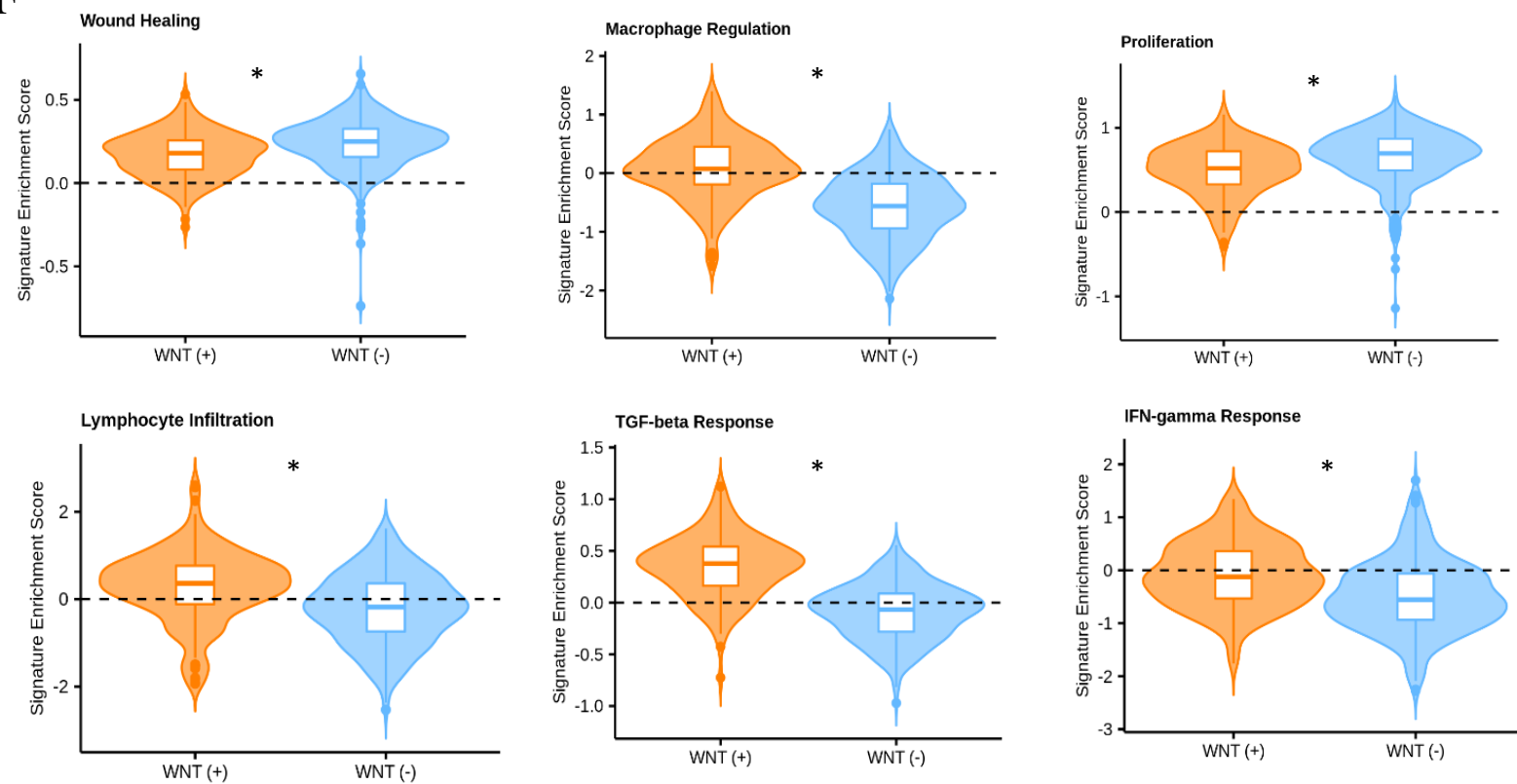

E

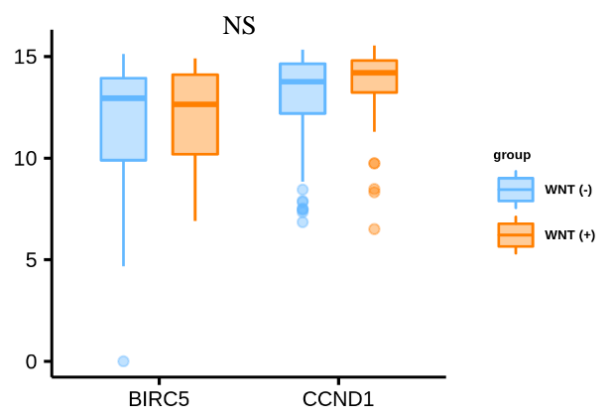

Supplement: Supplementary file 9 — Figure S8. A, Heatmap illustrating the expression levels of phosphorylated DDR markers in Wnt (+) and Wnt (–) tumors in the IRE cohort. The expression levels of DDR markers were calculated by multiplying staining intensity (0‐3) X the percentage of nuclear‐expressing tumor cells (final score range: 0‐300). B, Stacked bar chart summarizing the distribution of CRC TCGA subtypes in Wnt (+) and Wnt (–) cases. CIN: chromosomal instability, GS: genomically stable, MSI/POLE microsatellite instability/POLE. C, Violin plot illustrating the homologous repair deficiency (HRD) signature enrichment score in Wnt (+) and Wnt (–) cases in the TCGA study. D, Heatmap showing protein‐level expression of the Hippo transducers YAP and TAZ (IRE cohort) in the Wnt (+) and Wnt (–) groups. The expression of YAP/TAZ was calculated with the same method used for DDR markers. E, Box plot for the YAP/TAZ target genes BIRC5 and CCND1 (IRE cohort). NS: not significant. f Violin plots illustrating core immune signature scores (and the auxiliary proliferation signature) in the two compared subgroups (TCGA study). In the violin plots, NS: not significant, *: Mann‐Whitney test P < .05. [file CTM2-10-e199-s008.pdf]
