# Supplementary material for: Multicohort and cross‐platform validation of a prognostic Wnt signature in colorectal cancer
Source: Clin Transl Med. 2020 Dec 29;10(8):e199. doi: 10.1002/ctm2.199 (PMC7770515; doi:10.1002/ctm2.199)

A

	PFS HR [95%CI]	p-value
WNT (+) vs WNT (-)	2.35 [1.39-3.95]	0.001
Metastatic Sites (2-3 vs 1)	1.32 [0.80-2.15]	0.273
Surgery for Metastatic Disease (Yes vs No)	0.43 [0.26-0.69]	0.001
Sex (Male vs Female)	0.68 [0.42-1.11]	0.128
Age (continuous)	1.00 [0.98-1.02]	0.777

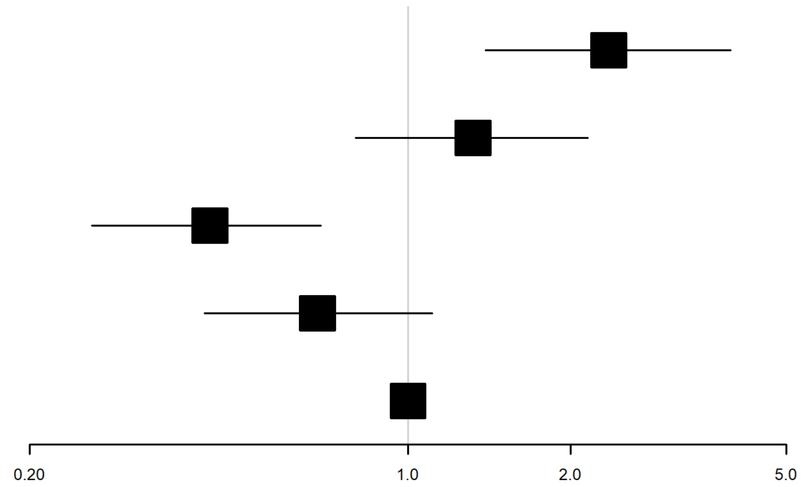

B

	OS HR [95%CI]	p-value
WNT (+) vs WNT (-)	1.75 [1.08-2.83]	0.024
Metastatic Sites (2-3 vs 1)	1.29 [0.79-2.11]	0.314
Surgery for Metastatic Disease (Yes vs No)	0.61 [0.37-0.99]	0.049
Sex (Male vs Female)	0.88 [0.54-1.44]	0.631
Age (continuous)	1.01 [0.99-1.03]	0.339

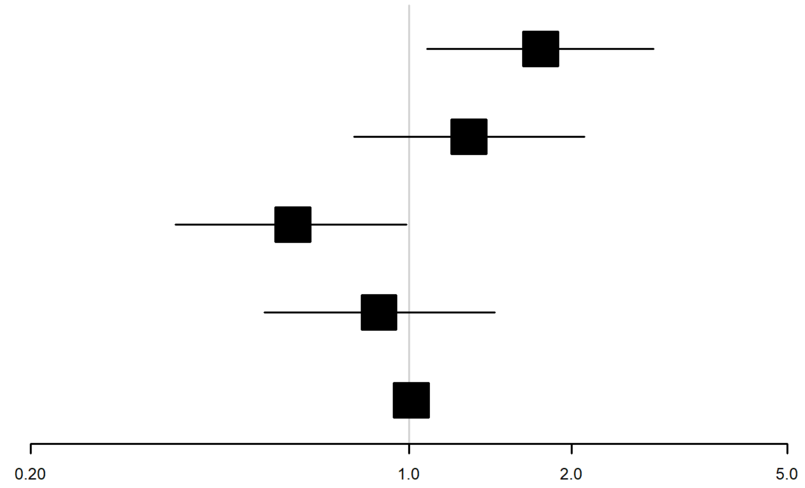

Supplement: Supplementary file 4 — Figure S3. Forest plots illustrating multivariate Cox regression analyses for progression‐free survival (PFS) (A) and overall survival (OS) (B) in the IRE cohort. [file CTM2-10-e199-s003.pdf]
