# Supplementary material for: Multicohort and cross‐platform validation of a prognostic Wnt signature in colorectal cancer
Source: Clin Transl Med. 2020 Dec 29;10(8):e199. doi: 10.1002/ctm2.199 (PMC7770515; doi:10.1002/ctm2.199)

# TCGA

| | OS HR [95%CI] | p-value |
|---|---|---|
| WNT (+) vs WNT (-) | 1.62 [1.10-2.38] | 0.015 |
| Age (above vs below median) | 2.99 [1.98-4.52] | <0.001 |
| Stage (4-3 vs 1-2) | 3.14 [2.13-4.65] | <0.001 |
| Primary disease location (colon vs rectum) | 1.36 [0.84-2.22] | 0.212 |
| Sex (female vs male) | 0.96 [0.66-1.39] | 0.811 |

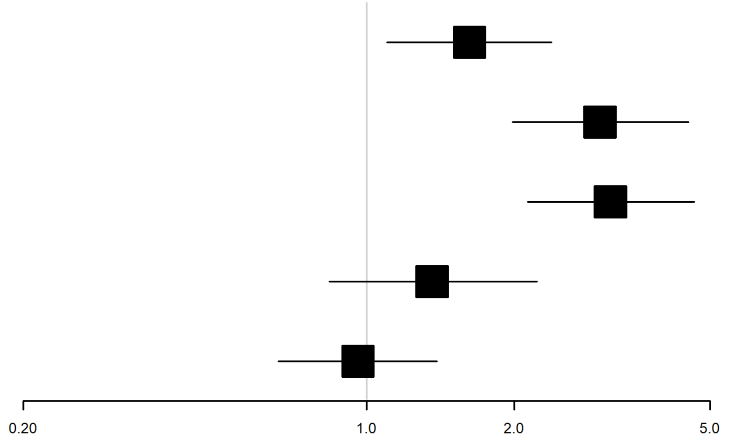

Supplement: Supplementary file 5 — Figure S4. Forest plot illustrating the multivariate Cox regression analysis for overall survival (OS) in the TCGA study. [file CTM2-10-e199-s004.pdf]
